# Worldwide Argus II implantation: recommendations to optimize patient outcomes

**DOI:** 10.1186/s12886-016-0225-1

**Published:** 2016-05-06

**Authors:** Devon H. Ghodasra, Adrienne Chen, J. Fernando Arevalo, David G. Birch, Kari Branham, Brian Coley, Gislin Dagnelie, Eugene de Juan, Robert G. Devenyi, Jessy D. Dorn, Andy Fisher, Duane R. Geruschat, Ninel Z. Gregori, Robert J. Greenberg, Paul Hahn, Allen C. Ho, Ashley Howson, Suber S. Huang, Raymond Iezzi, Naheed Khan, Byron L. Lam, Jennifer I. Lim, Kirsten G. Locke, Michelle Markowitz, Anne-Marie Ripley, Mark Rankin, Hannah Schimitzek, Fay Tripp, James D. Weiland, Jiong Yan, David N. Zacks, K. Thiran Jayasundera

**Affiliations:** Kellogg Eye Center, University of Michigan, Ann Arbor, MI USA; Wilmer Eye Institute, Johns Hopkins University School of Medicine, Baltimore, MD USA; Retina Foundation of the Southwest, Dallas, TX USA; Second Sight Medical Products, Inc., Sylmar, CA USA; University of California, San Francisco, San Francisco, CA USA; The University of Toronto, Toronto, Canada; Focal Point, Bristol, UK; Bascom Palmer Eye Institute, University of Miami, Miami, FL USA; Duke University Eye Center, Durham, NC USA; Wills Eye Hospital, Philadelphia, PA USA; Retina Center of Ohio, South Euclid, OH USA; Department of Ophthalmology, Mayo Clinic, Rochester, MN USA; University of Illinois at Chicago Eye and Ear Infirmary, Chicago, IL USA; Private Practice, Toronto, Canada; Canadian National Institute for the Blind, Toronto, Canada; Department of Ophthalmology, RWTH Aachen University, Aachen, Germany; Department of Ophthalmology, University of Southern California, Los Angeles, CA USA; Emory Eye Center, Atlanta, GA USA

## Abstract

**Background:**

A position paper based on the collective experiences of Argus II Retinal Prosthesis System investigators to review strategies to optimize outcomes in patients with retinitis pigmentosa undergoing retinal prosthesis implantation.

**Methods:**

Retinal surgeons, device programmers, and rehabilitation specialists from Europe, Canada, Middle East, and the United States were convened to the first international Argus II Investigator Meeting held in Ann Arbor, MI in March 2015. The recommendations from the collective experiences were collected. Factors associated with successful outcomes were determined.

**Results:**

Factors leading to successful outcomes begin with appropriate patient selection, expectation counseling, and preoperative retinal assessment. Challenges to surgical implantation include presence of staphyloma and inadequate Tenon’s capsule or conjunctiva. Modified surgical technique may reduce risks of complications such as hypotony and conjunctival erosion. Rehabilitation efforts and correlation with validated outcome measures following implantation are critical.

**Conclusions:**

Bringing together Argus II investigators allowed the identification of strategies to optimize patient outcomes. Establishing an on-line collaborative network will foster coordinated research efforts to advance outcome assessment and rehabilitation strategies.

## Background

Retinitis pigmentosa (RP) is a heterogeneous group of inherited retinal diseases affecting over one million individuals worldwide with an estimated prevalence of 1 in 4000 [1]. Though RP can be caused by mutations in any of over 190 genes, all lead to degeneration of the photoreceptor layer of the retina [2]. The relative preservation of inner retina has led to efforts to develop retinal prostheses to stimulate residual surviving tissue.

The Argus II Retinal Prosthesis System (Second Sight Medical Products, Inc., Sylmar, CA, USA) is a surgically implantable device designed to provide artificial vision to patients with outer retinal degenerative disease such as RP. It consists of an external video-processing unit that translates visual information from an eyeglass-mounted video camera into electrical signals. The implanted portion consists of a receiver coil that sends the electrical stimulus via a polymerized cable to a 60-electrode array that is implanted onto the retinal surface. Electrical stimulation of remaining retinal neurons evokes action potentials that travel through the optic nerve to the brain and elicit visual percepts. The device has been implanted in over 100 patients worldwide since receiving commercial approval in the European Union (CE Mark) in March 2011 and by the US Food and Drug Administration (FDA) in February 2013 [3]. The implant received medical device license approval by Health Canada in December 2014. In Saudi Arabia, it first received limited approval (restricted to the King Khaled Eye Specialist Hospital, Riyadh) in June 2012 and full approval in June 2015. Studies have shown promising results with visual function as well as improved performance on orientation and mobility tasks [4–8]. Longer-term studies have shown that the implant remains safe, and positive visual results can be sustained beyond 5 years of chronic use [9].

Since the Argus II prosthesis represents a novel paradigm for treatment of retinal disease, multidisciplinary review of early experiences is critical in recognizing potential challenges and refining current practices. The purpose of this manuscript is to summarize the recommendations to optimize patient outcomes with the Argus II device by analyzing the collective experience of investigators.

## Methods

The first international investigators’ meeting of retinal surgeons, device programmers, and rehabilitation specialists was convened in Ann Arbor, Michigan, USA in March 2015. The aim of the meeting was to share collective experiences with patient selection, surgical technique, outcome assessment, device programming issues, rehabilitation challenges, and future directions. Participants were asked to share factors contributing to optimal surgical outcomes; success with fitting, assessment, and rehabilitation; and patient satisfaction. This manuscript is a report of these collective experiences and recommendations to optimize patient outcomes with the Argus II system.

## Results and Discussion

### Patient selection

Retinal device implantation represents a significant investment from both healthcare payers as well as the patients themselves. While one study found Argus II to be a cost-effective intervention for RP patients compared to usual care, the device and implantation are associated with high initial costs [10]. In addition, frequent follow-up examinations and dedicated compliance with rehabilitation represent significant patient commitment.

Patient selection begins with a screening process typically conducted by low vision specialists and non-physician support staff. Since Argus II is currently only approved for subjects with profound RP, excluding patients with relatively good vision and incompatible diagnoses is important in reducing unnecessary examinations. The typical patient selection process involves a minimum of two clinical visits. Investigators recommend a full ophthalmologic evaluation including accurate documentation of visual function and anatomical assessment to determine factors that may affect successful implantation. Anterior segment evaluation should include notation of conjunctival or scleral thinning and phakic status. Dilated fundus examination encompasses documentation of any optic disc cupping, posterior vitreous detachment (PVD) status, presence of macular scar, posterior staphyloma, epiretinal membrane, and retinal breaks.

Ancillary testing includes optical coherence tomography and ocular ultrasonography looking for anatomic changes such as staphyloma or epiretinal membrane that may interfere with device placement. Additional pre-operative clinical visits include evaluation for general anesthesia readiness, review of consent forms, introduction to post-operative rehabilitation services, and counseling sessions to manage expectations.

The investigators identified the management of patient expectations as a critical component of the patient selection process. Counseling of patient expectations begins with the initial phone screening and continues through the post-operative period. Patients should be advised that the output from the device should be interpreted as an entirely new type of visual sensation rather than an attempt to restore previous vision. Most investigators felt that potential candidates could benefit from talking with selected past recipients of the device. Since family support throughout the pre-operative and post-operative is important, family members should be included in expectation counseling. Parallel conclusions can be drawn from experiences with pediatric cochlear implantation for hearing in which greatest success is achieved when the outcome matches or exceeds pre-operative expectations of the well-counseled family [11, 12]. Rehabilitation specialists strongly concur that past experiences with low vision and blindness rehabilitation, and emphasis on the importance of therapy prior to implantation is helpful in preparing patients for the significant commitment required after surgery. The investigators identified the following characteristics to be positively correlated with good outcome: reasonable expectations, supportive family members, existing blindness skills, patient familiarity with accessible technology for low vision and blindness, patient’s baseline functional abilities including general health, communication, and cognition.

Refining the criteria for patient selection in terms of the effect of patient age, presence of posterior staphyloma, and presence of severe outer retinal macular degeneration is still debated. A Phase 1 clinical trial is underway in Manchester, UK evaluating the feasibility of Argus II in severe dry age-related macular degeneration (AMD)/geographic atrophy [13]. There is ongoing investigation about factors that affect outcomes, including age. Investigators in Saudi Arabia and the Netherlands have examined the anatomical and functional outcome of patients less than 50 years old undergoing Argus II implantation. It has been hypothesized that younger patients have more preservation of the inner retinal layers, which could lead to better outcomes, but further research needs to be done.

Patients with abnormal posterior curvature of the eye such as staphyloma may not be ideal candidates for implantation since optimal signal transduction from the electrode array to the target cells requires good contact with the retinal surface. In vitro and in vivo studies have shown that retina-electrode distance significantly affects perceptual thresholds [14–16]. Proper centering of the array over the macula and creating close contact between the electrodes and retinal surface were identified as common challenges in implantation. Compression and puckering of the retina at the rim of the staphyloma, known as “snowplowing,” with resulting cystoid macular edema has also been observed by one investigator. Deformational force on the retina may cause cystoid macular edema in a manner similar to tractional forces causing vascular leakage and macular edema from conditions such as epiretinal membrane or vitreomacular traction. This phenomenon has been reported in one Argus II patient with staphyloma, but could conceivably arise in other conditions with abnormal posterior retinal curvature such as long axial length. Overall, retinal thickening with cystoid macular edema from all causes is not common and seen in less than 5 % of patients [17]. While some patients with less than ideal apposition have been pleased with their results, significant retina-array distance in staphyloma has also resulted in reduced sensitivity to electrical stimulation and in some cases electrophosphenes could not be generated at any stimulation level. A second retinal tack has been used to improve suboptimal array positioning. Alternatively, the renewed interest and successful outcomes of macular buckling for myopic traction maculopathy suggest a possible use for this technique in patients with staphyloma considering Argus implantation. The use of macular buckling or scleral slings, while not previously attempted in Argus patients, was discussed as a conceivable option to improve array apposition.

The Argus II directly stimulates the inner retina, and evidence of intact inner retinal function must be confirmed prior to implantation, particularly in patients with no light perception. This is typically performed with the dark-adapted photo flash test. In rare instances, when no conclusive determination of light perception is obtained with the photo-flash test, integrity of the optic nerve can be tested through Burian Allen contact lens electrical stimulation of the whole globe using a Digitimer D185 stimulator (Digitimer Ltd, Welwyn Garden City, United Kingdom) or similar instrument. Since some patients with certain types of RP have been shown to have corresponding inner retinal laminar abnormalities from retinal remodeling, optical coherence tomography (OCT) segmentation may have a role in patient selection [18]. Currently, OCT only provides information about inner retinal structure, and additional study is needed to determine how structure determines function in RP patients. In regards to outer retinal changes, some investigators have found poor implant functioning in some patients with severe outer retinal degeneration. Despite some cases of poor electrode functioning, patients can continue to show improved mobility and functional performance in the home environment using the Argus device. At present, pre-operative OCT is only used to identify staphyloma and epiretinal membranes that may affect implantation. Further study, however, is needed to investigate whether inner and outer retinal segmentation by OCT can be used to aid patient selection. Investigators note that part of the limitation is difficulties in obtaining adequate quality OCT scans due to lack of fixation and nystagmus. Efforts can be made to improve scans in these patients with coaching, patience, use of a Thornton ring, or in exceptional cases by performing a retrobulbar block.

### Surgical technique

Implanting the Argus II retinal prosthesis represents a new era in vitreoretinal surgery and with this comes a new set of challenges. The creation of this unique “silico-biologic” interface represents a refinement of several techniques already familiar to vitreoretinal surgeons.

If the patient is phakic, phacoemulsification with or without an intraocular lens (IOL) implantation should be performed one month prior or as a combined procedure with Argus II implantation. A 360° conjunctival peritomy is performed followed by isolation of the 4 recti muscles. The encircling band of the extraocular portion of the device is secured in a fashion similar to a scleral buckle. The electronics case and implant coil are carefully positioned in the superotemporal quadrant according to axial length-related tables and are sutured to the sclera through tabs located on the band. The investigators have identified this step as a critical component of the surgery since precisely measured external fixation is necessary for optimal positioning of the electrode array over the macula. Next, a 3-port pars plana vitrectomy with shave of peripheral vitreous is performed. The array and array cable is inserted through a 5.2 mm sclerotomy and secured to the retina-choroid-sclera with a custom-made titanium retinal tack.

Care must be taken throughout the procedure in handling the delicate electronics using only silicone tipped forceps. A patch graft is used to cover the suture tabs and the cable, and complete closure of the conjunctiva is critical. Standard postoperative steroid and antibiotic eye drops are administered and typical postoperative follow-up is indicated to monitor for adverse events.

### Adverse events

Surgery-associated adverse events including conjunctival erosion/dehiscence, hypotony, and endophthalmitis were found in 23, 13, and 10 % of patients, respectively, in the 3-year results of the Argus II trial [17]. An improved adverse event profile has since been reported following refinement of the procedure and device itself [19]. Nonetheless, retinal surgeons at the investigators meeting identified conjunctival erosion and hypotony as the most common surgical complications of implantation.

Hypotony following Argus II implantation typically arises from inadequate closure of sclerotomies, but less commonly may be due to damage to the ciliary body. Vigilant attention to closure, especially the superotemporal sclerotomy around the array cable, is critical. Sclerotomy closure is facilitated by proper initial wound construction. The sclerotomy for the array cable should be straight as opposed to a chevron or curved shape, which may cause cable and wound puckering. It should also be directed perpendicular to the sclera in order to prevent ciliary body detachment during insertion of the array into the vitreous cavity. During closure, mattress sutures with long scleral passes increase vector forces in re-apposing wound edges. The sutured wound should be dried and thoroughly checked for leakage. It should not be assumed that small amounts of oozing from the wound will resolve spontaneously. Other techniques which have been attempted, but where consensus regarding effectiveness has not been reached, include fluid-air exchange to increase vitreous plugging of the wound, partial thickness scleral flaps akin to trabeculectomy surgery, and the use of sealants such as corneal gel sealant or fibrin glue. When post-operative hypotony occurs, investigators agree that a short period of close follow-up with pressure patching can be sufficient if no other serious adverse signs are present. It is likely, however, that the patient may require reopening of the conjunctival/pericardial graft with further inspection and suturing of the sclerotomy. Persistent hypotony or other complications such as enlarging choroidal detachments and anterior chamber flattening should prompt return to the operating room for wound revision.

Conjunctival erosion is a second commonly identified complication of Argus II implantation and typically occurs over the raised profile of suture tabs. Several techniques were suggested to reduce the risk of this complication. The array cable and the anterior edge of the coil should be covered with processed pericardium or donor corneal graft. The manufacturer recommends Tenon’s membrane closure prior to conjunctival closure. Nylon suture is also preferred as the braided nature of Mersilene polyester sutures may contribute to erosion. Rotating knots posteriorly underneath suture tabs and leaving suture tails long may help with a lower profile knot that has a decreased risk of eroding conjunctiva. If conjunctival erosion is discovered post-operatively, topical antibiotics should be used until the patient can return to the operating room for closure. Wound revision should include adequately opening the area of eroded conjunctiva, thoroughly releasing any areas of traction, debriding or cauterizing areas of epithelialization, and re-covering exposed areas with pericardium or grafted conjunctiva. If the coil is well encapsulated and secure, some investigators suggest cutting off the suture tab.

Other less common complications associated with Argus II implantation include retinal detachment and endophthalmitis. To reduce the risk of retinal detachment, several surgeons recommended that triamcinolone acetonide be used to perform better shaving of the vitreous base in both the superotemporal and inferotemporal quadrants where the array and the tack, respectively, will be inserted. Since the vitreous cortex is very adherent in patients with RP, the hyaloid frequently cannot be detached anterior to the mid-periphery and excessive traction on the retina should be avoided. Similarly, macular epiretinal membranes should be peeled to improve contact between the electrodes and the retina. All agreed, however, that internal limiting membrane peeling is unnecessary and increases the risk of retinal holes in the macula. The investigators do not recommend prophylactic 360° laser retinopexy to the periphery. In cases of retinal tears or localized detachments observed post-operatively, laser retinopexy has been successfully used away from the array without change in electrode function. As in all intraocular surgeries, infectious endophthalmitis is a rare but potential devastating complication in Argus II implanted patients. With a high degree of suspicion and early treatment with intravitreal antibiotics, all cases of endophthalmitis in Argus II patients to date have been successfully resolved without the need to explant the device.

The incidence of adverse events continues to be equal to or lower than those reported in pre-market studies and in studies of glaucoma drainage devices [20]. Nevertheless, investigators suggest that patients should be educated about potential signs and symptoms of post-operative complications such as endophthalmitis and conjunctival erosion, as early detection is the key to maintaining successful outcomes.

### Device programming

Following surgical implantation, the Argus II device is fitted, or programmed, before the camera can be turned on. The aim of the first session is to gain an understanding of which electrodes on the array will be used, by first disabling individual electrodes whose resistance values are too high, and then performing a quick array scan to determine which electrodes on the array reliably yield percepts, or phosphenes, at different stimulation amplitudes. The second session involves measuring the perceptual threshold (i.e. the minimum current required to produce a percept the patient can see 50 % of the time) for electrodes that yielded a percept during array scanning. A video configuration file (VCF) is generated that defines how the video signal from the camera is mapped to the electrical signal for individual or groups of electrodes, and determines how many electrodes are stimulated simultaneously and at what frequency. Different image processing filters and VCF configurations are saved onto the patient’s VPU for use in different conditions: normal light conditions, contrast enhancement for low light conditions, and a setting for edge detection. The last programming step before turning the camera on compensates for the angle at which the array is placed on the retina during surgery.

Device programming can be an ongoing process as changes to either the array or a patient’s responses to electrode stimulation can occur. It is not uncommon for a patient to require several troubleshooting sessions to optimize the function of the device. Some challenges encountered to date include: decreasing sensitivity with ongoing stimulation, changes in perceptual thresholds, interference from spontaneous phosphenes, short duration of percepts, high thresholds or lack of thresholds, inadequate contrast, blurred contours/edges, poor alignment, inadequate radio frequency link between the glasses and the implant, overheating of the coil on the glasses, difficult battery insertion, poor eyeglass fit, and discomfort during stimulation. Post-surgical complications can also interfere with the programming process. Many of these challenges, however, can be remedied by an experienced programmer.

Some of the programming issues identified by investigators may be mitigated by the development of new programming software (the “Programming Assistant”) designed to streamline the programming process. This new software is intended to be more intuitive, to measure general sensitivity rather than specific thresholds, to simplify the camera alignment process, and to determine comfort under more real-world stimulation conditions. The aim is to reduce the programming time from 8 to 10 h, to 2 h or less. The new Programming Assistant will be tested in a small clinical trial, and if patient performance is not negatively impacted, approval will be sought to release the software in all markets. The glasses are also being redesigned for improved comfort.

To address other challenges encountered during the programming process, investigators had several suggestions. These included: a) Postponing the programming process until any surgical complications are resolved; b) Grouping electrodes together into groups of four (quads) or greater when percepts are not present after stimulating individual electrodes; c) Devising strategies to better counsel patients and their families regarding expectations for how their vision will change after device implantation. One center is developing a vision simulator to help families and rehabilitation specialists understand what a patient might experience with the Argus II system turned on; d) Implementing cross-training of the device programming and low vision rehabilitation teams so they can work together to improve the programming process; e) Offering targeted low vision rehabilitation to improve patient performance and satisfaction; f) Increasing our understanding of glare complaints by measuring photosensitivity and light perception.

### Outcome assessment

One striking, and perhaps unexpected, observation was that some performance measures, and especially performance of more complex, real-world tasks, did not necessarily correlate with a patient’s thresholds. In other words, having more electrodes with lower thresholds did not always translate to a better visual outcome or higher patient satisfaction, and patients with very different threshold levels (i.e. tending towards very high versus very low) can still have similar functional performance. This observation suggests it will be difficult to predict functional outcomes based on threshold levels alone.

There is currently no standardized measure to assess functional outcomes in patients with ultra-low vision. Because profound vision loss precludes conventional assessment of visual acuity, custom end-points were designed to measure functional outcomes for the Argus II clinical trial patients. Visual acuity was measured by three methods: grating visual acuity, square localization, and direction of motion [21]. Functional vision was evaluated with a “door task” and “line task.” Following discussions with FDA, the Functional Low-Vision Observer Rated Assessment (FLORA) was developed in response to an identified lack of qualified outcome measures to measure impacts on quality of life. However, this test may have limited utility as a standardized outcome measure due to its complexity and its reporting of subjective measures that are difficult to quantify [22]. A promising alternative for assessing outcomes and functional gains for Argus II patients is the Prosthetic Low Vision Rehab (PLoVR) questionnaire that was developed specifically for patients with ultra-low vision [23]. Difficulty ratings for a list of approximately 150 items/tasks relying on high/low contrast, lighting conditions, or form/movement were collected from ultra-low vision survey respondents and used to generate a logit scale where person ability and difficulty measures are plotted. This scale can then be used to measure changes in an individual’s visual ability over time. An adaptive version of the questionnaire has been recently developed and is being further refined [24].

### Rehabilitation challenges

Implantation of the Argus II into the eyes of patients with only bare or no light perception gives them access to a new visual input that needs to be integrated with any residual vision they possess, as well the low-vision and blindness skills they had previously developed. Rehabilitation aims to facilitate this integration in order to enhance quality of life and independence. The rehabilitation process consists of two major components: in-clinic rehabilitation and community rehabilitation. In the clinic, patients are taught the components of the Argus II system, how to manipulate the controls, as well as basic visual skills. Community-based rehabilitation is focused on visual integration into functional activities of daily living and refining existing blindness skills.

The Instructional kit provided by Second Sight consists of a collection of high contrast items, such as white shapes against a black background, and black and white plates and bowls. Most investigators use this kit to teach patients how to detect high contrast items of different shapes and configurations, and have found it useful and effective. Some investigators have added items such as cups of intermediate contrast (i.e. grey), canned foods, and medicine bottles. Patients who have used an in-home, scaled-down version of the kit have also reported on its utility. Overall, the investigators placed emphasis on working with objects that would help a patient improve functionality in their everyday lives. In some situations where patients have been unable to return to the clinic for regular rehabilitation visits, investigators have traveled to patients’ homes and report that patients have appreciated being able to work on aspects of visual function that are important for their day-to-day lives, such as seeing objects in their homes, or being able to go out and navigate in their neighborhoods. Working within the context of a familiar environment may also have a positive effect on a patient’s level of engagement and motivation. If the patient is employed, skills learned can also be incorporated into the work setting.

There are several device-related issues that may make the rehabilitation process more challenging. First, device fatigue and oversaturation may cause periods when having the device turned on is not useful. Second, patients can experience adaptation to the electrical stimulation, and as a result, percepts can get dimmer after extended device use. Third, new visual inputs from the Argus II system may be difficult to initially interpret. A major challenge of rehabilitation is determining how to integrate these new visual sensations with a patient’s low vision and blindness skills. It was suggested that an optimal strategy may be to teach patients to retain their existing blindness skills such as auditory and tactile function, and to supplement these with the vision provided by the Argus II.

In addition to their critical role in teaching patients how to optimally use and integrate their new visual inputs into their daily lives, the investigators felt that rehabilitation specialists could also make important contributions to evaluating potential patients, both in terms of looking for possible barriers to successful rehabilitation, and managing the expectations of patients and their families as to the degree of vision the patient will experience. Pre-surgical interactions with the rehabilitation specialist on the team would also help to build interpersonal relationships that could facilitate the rehabilitation process, and the specialist could also stress the importance of coaching at home and family participation.

## Conclusion

The gathering of retinal surgeons, device programmers, rehabilitation specialists, and industry representatives allowed a comprehensive review of the Argus II implantation and rehabilitation process. Early experience with the Argus II implant has delivered promising results and highlighted new challenges. The meeting of investigators from around the world also allowed the coordination of future research efforts. A collaborative listserve will be established and several interesting topics of further study were identified. These included the examination of longitudinal changes in electrical thresholds in implanted patients, the study of cross-modal activity on brain MRI as a marker for Argus II function, the potential for retinal recordings directly from the electrode array, and the feasibility of implantation for age-related macular degeneration. The present time represents a critical juncture for retinal prosthetic systems- an opportunity to make adjustments in techniques and procedures that will drive future outcomes.

## Ethics approval and consent to participate

Not applicable.

## Consent for publication

Not applicable.

## Availability of data and materials

Not applicable.

## Abbreviations

FDA: Food and Drug Administration
OCT: optical coherence tomography
VCF: video configuration file
RP: retinitis pigmentosa
