# Erratum to: Worldwide Argus II implantation: recommendations to optimize patient outcomes

**DOI:** 10.1186/s12886-016-0259-4

**Published:** 2016-07-29

**Authors:** Devon H. Ghodasra, Adrienne Chen, J. Fernando Arevalo, David G. Birch, Kari Branham, Brian Coley, Gislin Dagnelie, Eugene de Juan, Robert G. Devenyi, Jessy D. Dorn, Andy Fisher, Duane R. Geruschat, Ninel Z. Gregori, Robert J. Greenberg, Paul Hahn, Allen C. Ho, Ashley Howson, Suber S. Huang, Raymond Iezzi, Naheed Khan, Byron L. Lam, Jennifer I. Lim, Kirsten G. Locke, Michelle Markowitz, Anne-Marie Ripley, Mark Rankin, Hannah Schimitzek, Fay Tripp, James D. Weiland, Jiong Yan, David N. Zacks, K. Thiran Jayasundera

**Affiliations:** 1 Kellogg Eye Center, University of Michigan, Ann Arbor, MI USA; 2 Retina Division, Wilmer Eye Institute, Johns Hopkins University School of Medicine, Baltimore, MD USA; 3 Wilmer Eye Institute, Johns Hopkins University School of Medicine, Baltimore, MD USA; 4 Vitreoretinal Division, King Khaled Eye Specialist Hospital, Riyadh, Kingdom of Saudi Arabia; 5 Retina Foundation of the Southwest, Dallas, TX USA; 6 Second Sight Medical Products, Inc., Sylmar, CA USA; 7 University of California, San Francisco, CA USA; 8 The University of Toronto, Toronto, Canada; 9 Focal Point, Bristol, UK; 10 Bascom Palmer Eye Institute, University of Miami, Miami, FL USA; 11 Duke University Eye Center, Durham, NC USA; 12 Wills Eye Hospital, Philadelphia, PA USA; 13 Retina Center of Ohio, South Euclid, OH USA; 14 Department of Ophthalmology, Mayo Clinic, Rochester, MN USA; 15 University of Illinois at Chicago Eye and Ear Infirmary, Chicago, IL USA; 16 Private Practice, Toronto, Canada; 17 Canadian National Institute for the Blind, Toronto, Canada; 18 Department of Ophthalmology, RWTH Aachen University, Aachen, Germany; 19 Department of Ophthalmology, University of Southern California, Los Anges, CA USA; 20 Emory Eye Center, Atlanta, GA USA

Unfortunately, the original version of this article [1] contained an error. The second affiliation of Dr. J Fernando was not included. The correct version which is both affiliations is included here.

Retina Division, Wilmer Eye Institute, Johns Hopkins University School of Medicine, Baltimore, MD, USA, and the Vitreoretinal Division, King Khaled Eye Specialist Hospital, Riyadh, Kingdom of Saudi Arabia.
